# Genome-wide association and genomic prediction for resistance to southern corn rust in DH and testcross populations

**DOI:** 10.3389/fpls.2023.1109116

**Published:** 2023-01-26

**Authors:** Jinlong Li, Dehe Cheng, Shuwei Guo, Chen Chen, Yuwen Wang, Yu Zhong, Xiaolong Qi, Zongkai Liu, Dong Wang, Yuandong Wang, Wenxin Liu, Chenxu Liu, Shaojiang Chen

**Affiliations:** ^1^ National Maize Improvement Center of China, Key Laboratory of Crop Heterosis and Utilization Ministry of Education (MOE), China Agricultural University, Beijing, China; ^2^ Maize Research Institute, Beijing Academy of Agriculture and Forestry Sciences, Beijing, China

**Keywords:** maize, southern corn rust resistance, genome-wide association study, genomic prediction, models

## Abstract

Southern corn rust (SCR), caused by *Puccinia polysora* Underw, is a destructive disease that can severely reduce grain yield in maize (*Zea mays* L.). Owing to *P. polysora* being multi-racial, it is very important to explore more resistance genes and develop more efficient selection approaches in maize breeding programs. Here, four Doubled Haploid (DH) populations with 384 accessions originated from selected parents and their 903 testcross hybrids were used to perform genome-wide association (GWAS). Three GWAS processes included the additive model in the DH panel, additive and dominant models in the hybrid panel. As a result, five loci were detected on chromosomes 1, 7, 8, 8, and 10, with *P*-values ranging from 4.83×10^-7^ to 2.46×10^-41^. In all association analyses, a highly significant locus on chromosome 10 was detected, which was tight chained with the known SCR resistance gene *RPPC* and *RPPK*. Genomic prediction (GP), has been proven to be effective in plant breeding. In our study, several models were performed to explore predictive ability in hybrid populations for SCR resistance, including extended GBLUP with different genetic matrices, maker based prediction models, and mixed models with QTL as fixed factors. For GBLUP models, the prediction accuracies ranged from 0.56-0.60. Compared with traditional prediction only with additive effect, prediction ability was significantly improved by adding additive-by-additive effect (*P*-value< 0.05). For maker based models, the accuracy of BayesA and BayesB was 0.65, 8% higher than other models (i.e., RRBLUP, BRR, BL, BayesC). Finally, by adding QTL into the mixed linear prediction model, the accuracy can be further improved to 0.67, especially for the G_A model, the prediction performance can be increased by 11.67%. The prediction accuracy of the BayesB model can be further improved significantly by adding QTL information (*P*-value< 0.05). This study will provide important valuable information for understanding the genetic architecture and the application of GP for SCR in maize breeding.

## 1 Introduction

Southern corn rust (SCR) caused by *Puccinia polysora* Underw, is one of the most devastating maize diseases, widely distributed in Asia, America, Africa and other major corn production areas (Sun et al., 2021). SCR was first reported by Underwood in 1897 in the USA (Underwood, 1897) and observed in most tropical and temperate maize-growing areas of the world in subsequent decades (Orian, 1954; Duan and He, 1984). The invasiveness of leaves and stems of maize resulted in yield losses of up to 50% (Rhind et al., 1952; Liu and Wang, 1999). The wide distribution, long-distance migration, multiple physiological races and fast evolution made SCR difficult to be controlled, causing great grain yield losses (Sun et al., 2021). With climate change, SCR tends to further increase and expand to higher latitudes regions (Ramirez-Cabral et al., 2017).

The breeding of SCR resistant varieties is very important for disease management, which poses challenges for breeders. In China, several main cultivated corn varieties, such as Zhengdan958, Xundan20 and Xianyu335, have been identified to be susceptible to SCR (Yuan et al., 2010). Indeed, Wang et al. (Wang et al., 2006) investigated the resistance of 178 corn varieties to SCR, and reported that only 14% of varieties were highly resistant to SCR. On the other hand, Zhou et al. (Zhou et al., 2017) identified several highly resistant germplasms, such as DH02, Zheng39, T2 and JH3372. In addition, some inbred lines such as AFR024 (Storey and Howland, 1957), Qi319 (Chen et al., 2004), CML470 (Yao et al., 2013), J2416K (Wang et al., 2020) were also found to be resistant germplasm. The discovery of these germplasms not only improved the variety resistance by breeding, but also provided the basis for gene detection.

Based on geographic distribution, more than 10 physiological races of *P*. *polysora* have been identified, including EA.1, EA.2, EA.3, and PP.3-PP.9 (Ryland and Storey, 1955; Storey and Howland, 1957; Robert, 1962; Ullstrup, 1965). Owing to the rapid development of genetics, so far, several unique, major, race-specific SCR-resistance genes have been reported. *Rpp1*, a fully dominant gene, was identified as a resistance gene to *P. polysora* races EA.1 and EA.3; *Rpp2*, a partially dominant gene closely linked with *Rpp1*, was resistant to races EA.1, EA.2, and EA.3; *Rpp9*, a single dominant gene on 10.01 bin, was resistant to race PP.9 (Storey and Howland, 1959; Storey and Howland, 1967). It is noteworthy that *Rpp9* is closely linked, with a genetic distance of 1.5 cM, to a common rust resistance gene *rp1*, but its genomic location had not been confirmed (Ullstrup, 1965). In recent years, more resistance loci on chromosome 10 have been detected, including *RppP25* (Liu et al., 2003), *RppQ* (Chen et al., 2004), *RppD* (Zhang et al., 2009), *RppC* (Yao et al., 2013), *Rpp12* (Zhang, 2013), *RppS* (Wu et al., 2015), *RppM* (Wang et al., 2020), *qSCR6.01* (Lu et al., 2020), *RppCML496* (Lv et al., 2021), *RppK* (Chen et al., 2022).

Genome-wide association study (GWAS), which is based on genetic linkage disequilibrium (LD) in a panel including a large number of genotypes representing broadly natural variations, has been used as an alternative approach for exploring the molecular basis and identifying SNPs of complex quantitative traits (Yu and Buckler, 2006). In maize, GWAS has been successfully utilized to identify numerous candidate loci/genes controlling disease resistance, such as head smut (Wang et al., 2012) common rust (Kibe et al., 2020; Ren et al., 2021), rough dwarf (Zhao et al., 2021), ear rot (Guo et al., 2020), gray leaf spot (Mammadov et al., 2015), etc. For SCR, eight SNPs were identified as significant loci using GWAS with a panel of 164 maize inbred lines in previous studies (Souza Camacho et al., 2019). The results of these studies provide valuable information on understanding the mechanism of disease resistance and breeding superior varieties.

Genomic prediction (GP), also known as genomic selection (GS), is a technology to predict the performance of plants without phenotyping, and has been proven to be effective in plant breeding (Meuwissen et al., 2001; Cerrudo et al., 2018). Gowda et al. (Gowda et al., 2015) successfully modeled the resistance of lethal necrosis disease in tropical maize germplasm with ridge regression best linear unbiased prediction (RRBLUP). For common rust, GP accuracies observed in the GWAS panel and Doubled Haploid (DH) population were 0.61 and 0.51 (Ren et al., 2021). For goss’s wilt, the GP model was trained with an accuracy of 0.69 (Cooper et al., 2019). However, in maize hybrids, there are few cases of genomic prediction for disease resistance.

In this study, four DH populations with 384 accessions and their testcross hybrids with 903 accessions were used to perform GWAS and GP analyses for SCR resistance. The objectives of the current study were to (1) detect the significantly associated SNPs, and major QTL conferring SCR resistance; (2) predict SCR resistance trait with different GBLUP models; (3) test the predictive power of different marker-based models for resistance trait; and (4) estimate the GP accuracies using models with QTL information.

## 2 Materials and methods

### 2.1 Plant materials

A total of 384 DH lines belonging to four DH populations were developed from four elite inbred lines (**Table 1**) in BeiJing (N40°08’ E116°10’) in 2017. The founders of the four DH populations were C783 × C229, C783 × UH306, C783 × EH, C229 × UH306, respectively, and we named them as POP1-4. The quantities of DH lines in POP1-4 are 66, 107, 127, and 77, respectively. Then, we testcross each population with three testers, yielding a total of 903 hybrids (**Table 1**). Thus, the hybrid population is divided into 12 subgroups with quantities ranging from 40 to 119.

**Table 1:** Summary of extended GBLUP models with different relationship matrices.

Model	Additive	Dominance	Epistasis
G_A	**Ga**		
G_D		**Gd**	
G_A_D	**Ga**	**Gd**	
G_A_AA	**Ga**		**Ga#Ga**
G_A_AD	**Ga**		**Ga#Gd**
G_A_DD	**Ga**		**Gd#Gd**
G_A_D_E	**Ga**	**Gd**	**Ga#Ga, Ga#Gd, Gd#Gd**

**#,** Hadamard products to compute the epistatic matrices terms.

To test the SCR infection levels of the accessions, we planted the DH lines and hybrids in Huang-Huai-Hai summer corn planting region in China for phenotypic identification. This region is the main area where SCR occurs in China due to high temperature and rainy summer. The DHs were planted in Jinan (N37°42’ E117°27’) in 2021, Xinxiang (N35°9’ E113°47’) in 2018 and 2021; the hybrids were planted in Jinan in 2021, Jining (N35°6’ E116°31’) in 2020 and 2021, Xinxiang in 2020. We used the augmented experimental design, setting every 20 accessions as a block. Each block consisted of 19 rows and standard accessions were planted in random order. For the DHs, the standard accession was a susceptible inbred line C116A. For the hybrids, the standard accession was a susceptible commercial hybrids ZhengDan958. In the field, each accession was planted in a one-row plot for DHs and a two-row plot for hybrids at a spacing with 0.6 x 0.25 m spacing (66,000 plants per hectare).

### 2.2 Southern corn rust resistance score (SCRRS) collection

**Figure 1:**
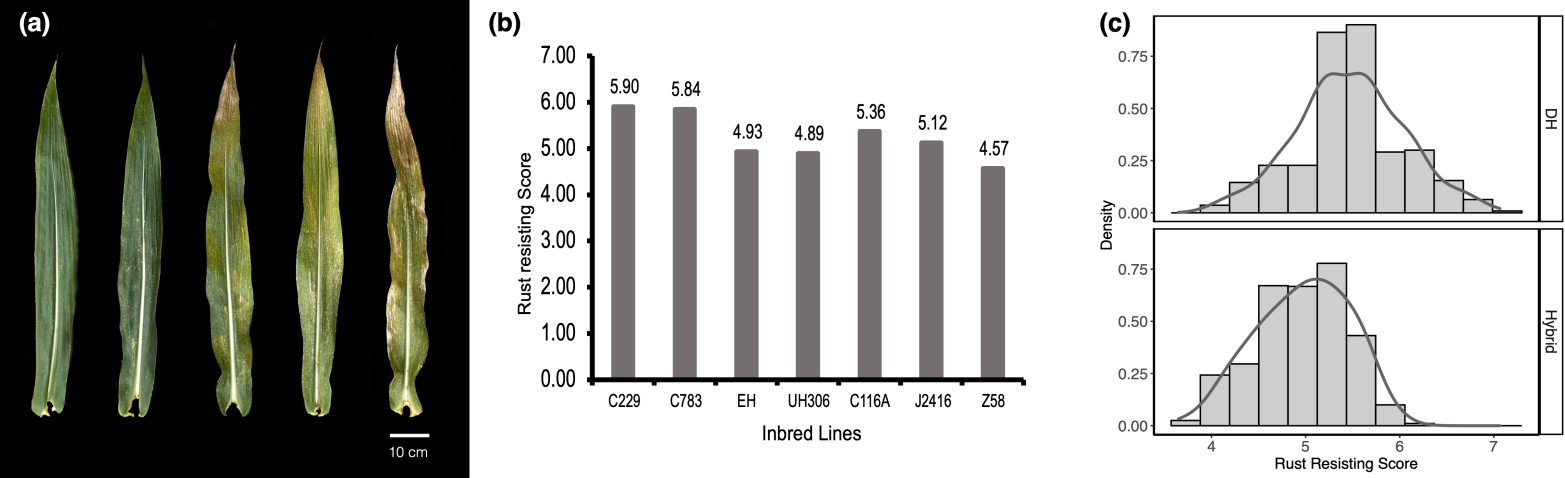
Southern Corn Rust Resistance Score (SCRRS) and its distribution. **(A)** The manifestation of susceptible leaves, the SCRRS of leaves were 9, 7, 5, 3, 1 from left to right. **(B)** the SCRRSs in DH founders and testers. **(C)** The distribution of SCRRS in DH (top) and hybrid (bottom) populations.

SCRRS for each accession was visually collected from the leaf area covered by lesions at 4 weeks after flowering (**Figure 1A**). A rating scale of 1 corresponds to severe infection covering > 75% of the leaf surface, 3 corresponds to moderate-to-severe infection covering 50–75% of the leaf surface, 5 corresponds to moderate infection covering 25–50% of the leaf surface, 7 corresponds to weak to moderate infection covering 10–25% of the leaf surface, and 9 corresponds to high resistance covering 0–10% of the leaf surface (Ren et al., 2021).

### 2.3 Phenotypic data analysis

The raw phenotypic data were analyzed using the linear mixed model with an R add-on package “lme4” (Bates et al., 2014). Best linear unbiased predictors (BLUPs) were calculated for DHs and hybrids. In the model,


$$y_{ij}=\mu +g_{i}+l_{j}+g_{i}\times l_{j}+\epsilon$$

where, *y_ij_* is the mean phenotypic value of the *i*th DH or hybrid in the *j*th environment;

*μ* is the overall mean of the trait; *g_i_* is the random effect of the *i*th accession; *l_j_* is the random effect of the *j*th environment; *g_i_* ×*l_j_* is the random interaction effect between the *i*th accession and the *j*th environment; and ϵ is the random error.

Heritability was calculated using variance components estimated from the above model. The following equation was used to estimate heritability on an individual plot basis,


$$H^{2}=\frac{V_{g}}{V_{g}+\frac{V_{e}}{l}}$$

Where *V_g_* is the genotypic variance component, *V_e_* is the error variance, and *l* is the number of environments.

### 2.4 Genotyping and genotypic data analysis

Young leaves of all the DHs and the tester lines were sampled for DNA extraction using the CTAB method (Porebski et al., 1997). Then, genotyping was conducted using the Maize-6H-60K SNP chip (Tian et al., 2021). SNPs with minor allele frequency (MAF) > 0.05 and per locus missing rate< 0.1 were filtered out using plink 1.90 (http://www.cog-genomics.org/plink2/). The genotypes of hybrids were obtained with the cleaned SNPs (N=34,037) of DHs and testers using TASSEL V5.2 software (Bradbury et al., 2007). Pairwise measures of linkage disequilibrium (LD) were performed to analyze the squared allele‐frequency correlation coefficient (r^2^) between two loci using plink software. Only SNPs with a MAF > 0.05 and less than 0.1 missing data were used to estimate LD. Principal component analysis (PCA) was used to assess the level of genetic structure using TASSEL software.

### 2.5 Genome wide association study

Genome wide association analysis was performed with the BLUPs obtained from the combined analysis for the DHs and hybrids. A Fixed and Random Model Circulating Probability Unification (FarmCPU) method, as proposed by Liu et al. (Liu et al., 2016) was applied in GAPIT V3 software (Wang and Zhang, 2021). Two genetic models, additive and dominant, are used for the hybrids panel, and only the additive model was used for the DH panel. Under the additive model, homozygous genotypes with recessive allele combinations were coded as 0, homozygous genotypes with dominant allele combinations are coded as 2, and heterozygous genotypes were coded as 1. Under the dominant model, both types of homozygous genotypes are coded as 0 and heterozygous genotypes were coded as 1. The Bonferroni testing was used to determine the genome-wide significance thresholds (0.05/34,034 = 1.47 × 10^−6^), where 34,037 is the total number of SNP markers (Holm, 1979). Markers whose *P-*values passed the threshold were identified as candidate loci. Unlike natural material populations, such as the artificial DH population or testcross hybrid population, which had a high LD level, our candidate intervals were selected according to LD decay and LD block. Makers with a physical distance of<20 Mb and in high LD (r^2^ ≥ 0.8) were considered to mark the same genomic region (Mayer et al., 2020). The corresponding candidate region was described by the positions of the first and last maker, respectively.

### 2.6 Genomic prediction

The Genomic prediction was performed for the hybrid panel with three conditions, including 1) extended GBLUP models, 2) maker based prediction methods, and 3) prediction models with QTL calculated by GWAS as fixed effects.

For extended GBLUP models, which comprised additive (**G_a_**), dominant (**G_d_**) and epistatic relationship matrices. **G_a_** and **G_d_** matrices were calculated using the “sommer” package in R (Covarrubias-Pazaran, 2016). The epistatic matrices terms were computed using Hadamard products (i.e., cell-by-cell product denoted as **“#”**) of the following form: (i) additive-by-additive interactions (**G_a_#G_a_**); (ii) dominance-by-dominance interactions (**G_d_#G_d_**); and (iii) additive-by-dominance interactions (**G_a_#G_d_**), respectively (Muñoz et al., 2014). In total, six GBLUP models were used in this study (**Table 1**). The programs were implemented in the “BGLR” package in R (Pérez and De Los Campos, 2014). The extended GBLUP models can be described as

Model(G_A): **y=1*_n_* μ+*G_a_u_a_* +ϵ**
Model(G_D): **y=1*_n_* μ+*G_d_u_d_* +ϵ**
Model(G_A_D): **y=1*_n_* μ+*G_a_u_a_* +*G_d_u_d_* +ϵ**
Model(G_A_AA): **y=1*_n_* μ+*G_a_u_a_* +*G_aa_u_aa_* +ϵ**
Model(G_A_AD): **y=1*_n_* μ+*G_a_u_a_* +*G_ad_u_ad_* +ϵ**
Model(G_A_DD): **y=1*_n_* μ+*G_a_u_a_* +*G_dd_u_dd_* +ϵ**
Model(G_A_D_E): **y=1*_n_* μ+*G_a_u_a_* +*G_d_u_d_* +*G_aa_u_aa_* +*G_ad_u_ad_* +*G_dd_u_dd_* +ϵ**

where ***y*** is the vector of phenotypic data; ***1_n_*** is the n-dimensional vector of ones; *μ* is the overall mean; ***u_a,_ u_d,_ u_aa,_ u_ad,_ u_dd_*** are the vectors of random effects for additive, dominant, additive-by-additive, additive-by-dominance and dominance-by-dominance effects assumed to obey the normal distributions N(0, ${\text{G}}_{\text{a}}\sigma_{\text{a}}^{2}$ ), N(0, ${\text{G}}_{\text{d}}\sigma_{\text{d}}^{2}$ ), N(0, ${\text{G}}_{\text{aa}}\sigma_{\text{aa}}^{2}$ ), N(0, ${\text{G}}_{\text{ad}}\sigma_{\text{ad}}^{2}$ ) and N(0, ${\text{G}}_{\text{dd}}\sigma_{\text{dd}}^{2}$ ), respectively; ***G_a_***, ***G_d_***, ***G_aa_***, ***G_ad_*** and ***G_dd_*** are the genomic relationship matrices corresponding to additive, dominance, additive-by-additive, additive-by-dominance and dominance-by-dominance genotypic values, respectively.

We also performed maker based prediction models including RRBLUP (Whittaker et al., 2000), BRR (Pérez and De Los Campos, 2014), BL (Park and Casella, 2008), BayesA-C (Meuwissen et al., 2001b). The RRBLUP method is based on a restricted maximum likelihood (REML) approach to ridge regression, we performed it by R package “rrBLUP” (Endelman, 2011). Meanwhile, we also used Bayes-based methods to fit models, containing different prior densities, i.e., Gaussian (BRR), Double exponential (BL), Scaled-t (BayesA), Scaled-t mixture (BayesB), Gaussian mixture (BayesC) in BGLR package (Pérez and De Los Campos, 2014). The basic model is,


$$y=1_{n}\text{μ}+Z\alpha +\epsilon$$

where ***y*** is the vector of phenotypes; ***1_n_*** is the n-dimensional vector of ones; *μ* is the overall mean,; ***α*** is a vector of random regression coefficients of all the marker effects; ***Z*** is an genotypic matrix for markers; and ***ϵ*** is a vector of residuals. The alternative methods discussed here differ primarily in their specific prior used for ***α***. For RRBLUP, α~N(0, $\text{I}\sigma_{\text{a}}^{2}$ ) and $\sigma_{\text{a}}^{2}$ has a scaled inverse chi-square distribution. For BayesA, the unconditional distributions of the marker effects follow identical and independent univariate *t* distributions, each with mean zero. BayesB employs a mixture distribution that includes a point of mass at zero and a univariate scaled *t* distribution. The assumption of BayesC is that each marker effect is zero with probability π and follows a univariate normal distribution with probability (1 − π) having mean zero and variance $\sigma_{j}^{2}$ , which has a scaled inverse chi-square distribution.

To further improve the prediction ability, we added QTL into the mixed linear model as fixed factors. Two representative models were selected, namely G_A and BayesB. We added additive localization maker and dominant localization maker obtained by GWAS into the model separately or together, including G_A_qa (G_A with additive GWAS SNPs), G_A_qd (G_A with dominant GWAS SNPs), G_A_qad (G_A with additive and dominant GWAS SNPs), BayesB_qa (BayesB with additive GWAS SNPs), BayesB_qd (BayesB with dominant GWAS SNPs), BayesB_qad (BayesB with additive and dominant GWAS SNPs). When the prediction was performed with additive QTL, homozygous genotypes with recessive allele combinations were coded as 0, homozygous genotypes with dominant allele combinations were coded as 2, and heterozygous genotypes were coded as 1. When the prediction was performed with dominant QTL, both types of homozygous genotypes were coded as 0 and heterozygous genotypes were coded as 1. The models can be described as,

Model(G_A_qa): **y=*X_QTLa_β_a_* +*G_a_u_a_* +ϵ**
Model(G_A_qd): **y=*X_QTLd_β_d_* +*G_a_u_a_* +ϵ**
Model(G_A_qad): **y=*X_QTLad_β_ad_* +*G_a_u_a_* +ϵ**
Model(BayesB_qa): **y=*X_QTLa_β_a_* +Z*α_a_* +ϵ**
Model(BayesB_qd): **y=*X_QTLd_β_d_* +Z*α_d_* +ϵ**
Model(BayesB_qad): **y=*X_QTLad_β_ad_* +Z*α_ad_* +ϵ**

where ***y*** is the vector of phenotypes; ***X_QTLa_, X_QTLd_, X_QTLad_*** are incidence matrices of additive localization makers, dominant localization makers and both, respectively; ***β_a_, β_d_*** and ***β_ad_*** are vectors of fixed effects for ***X_QTLa_, X_QTLd_*** and ***X_QTLad_***, respectively; ***G_a_*** is the genomic relationship matrix corresponding to additive genotypic values; ***Z*** is a genotypic matrix for all markers; ***α*** is a vector of random regression coefficients of all the marker effects; and ***ϵ*** is a vector of residuals.

In this study, we used a five-fold cross validation approach to assess the ability of the tested GP models. Prediction accuracy was quantified using two methods, 1) the Pearson correlation between the input trait values and the genomic estimated breeding values (GEBVs) predicted from a given GS model evaluated in the test set, 2) the number of top 20% accessions intersections selected by GEBVs and true values derived by the total number of accessions in the test set. The process was repeated 100 times to eliminate the prediction error.

### 2.7 Statistical analysis

Data analysis was carried out with R software (Version 3.6.2). Microsoft Excel for Mac (Version 16.50) was used for collation of phenotypic data. Tukey’s test and Students’ t-test were performed to assess the significance of differences between values, and *P* < 0.05 was considered to be statistically significant.

## 3 Results

### 3.1 Phenotypic variations and heritability

We evaluated the SCRRS in 384 DH lines and 903 hybrids under three and four environments, respectively. The results indicated that there were abundant phenotypic variations within each panel (**Figures 1B, C**). The descriptive statistics for each population are presented in **Table 2**. For DH founders, C229 and UH306 showed the highest and lowest SCRRSs, which were 5.90 and 4.89 respectively. In the DH panel, the SCRRS ranged from 4.10–7.06, and POP1 showed significantly high resistance to SCR (Tukey-test, *P<*0.05), with the mean SCRRS was 6.02. In the hybrid panel, the scores ranged from 3.65 to 6.08, with a mean of 4.97. The most resistant subgroup was POP2/C229, with the mean SCRRS was 5.39. In particular, the DHs and hybrids were planted at different locations, so we didn’t make a comparison between the two panels. The broad-sense heritability (*H^2^*) analysis revealed that the *H^2^* in the DH panel and the hybrid panel were 0.64 and 0.54, respectively, suggesting that the phenotypic variation in the two panels was genetically controlled.

**Table 2:** Descriptive statistics, variance components, and broad-sense heritability (*H^2^*) of southern corn rust resistance.

Subgroup	DH Founder	Tester	NO.	Min	Max	Mean	SD	Tukey-test	Variance components			*H^2^*
									$\sigma_{\text{g}}^{2}$	$\sigma_{\text{ge}}^{2}$	$\sigma_{\text{e}}^{2}$	
POP1	C783, C229		66	4.73	7.06	6.04	0.49	c	0.98	0.16	1.62	0.64
POP2	C783, UH306		107	4.73	6.75	5.59	0.44	b				
POP3	C783, EH		127	4.1	6.13	5.28	0.46	a				
POP4	C229, UH306		77	4.1	6.56	5.17	0.57	a				
**DH Mean**			94	4.42	6.63	5.52	0.49					
POP1/C116A	C783, C229	C116A	51	4.11	5.89	5.09	0.58	cd	0.64	1.14	2.16	0.54
POP1/EH	C783, C229	EH	40	3.89	5.61	4.83	0.41	bc				
POP1/J2416	C783, C229	J2416	52	3.89	6.08	5.01	0.56	c				
POP2/C116A	C783, UH306	C116A	87	3.89	5.58	4.7	0.37	ab				
POP2/C229	C783, UH306	C229	80	4.14	5.85	5.39	0.31	e				
POP2/Z58	C783, UH306	Z58	90	4.11	5.86	5.00	0.38	c				
POP3/C116A	C783, EH	C116A	111	3.67	5.86	4.67	0.45	ab				
POP3/C229	C783, EH	C229	102	4.21	5.85	5.31	0.34	de				
POP3/Z58	C783, EH	Z58	119	3.65	5.86	4.93	0.44	c				
POP4/C116A	C229, UH306	C116A	51	4.17	5.64	4.95	0.45	c				
POP4/C783	C229, UH306	C783	59	4.77	5.62	5.33	0.26	de				
POP4/J2416	C229, UH306	J2416	61	3.68	5.4	4.48	0.41	a				
**Hybrid Mean**			75	4.02	5.76	4.97	0.41					

NO., the accession number of each subgroup.

SD, standard deviation.

Tukey-test, significant at P< 0.05. $\sigma_{\text{g}}^{2}$ , genotypic variance. $\sigma_{\text{ge}}^{2}$ , genotype × environment interaction variance. $\sigma_{\text{e}}^{2}$ , error variance.

H^2^, broad-sense heritability.

### 3.2 Genotype and population structure analysis

**Figure 2:**
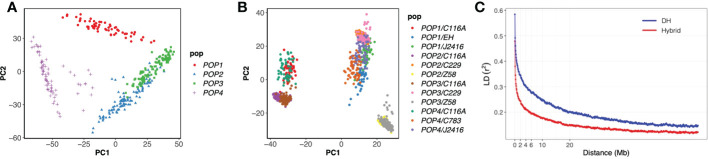
Analysis of genetic structure in the DH and hybrid panels. **(A)** the principal component analysis for the DH panel. **(B)** the principal component analysis for the hybrid panel. **(C)** Linkage disequilibrium decay in the two populations.

After marker quality control (see Materials and Methods), 34,037 SNP markers for 384 DH genotypes were available for further analysis. The 903 hybrid genotypes were imputed by their parents. The molecular diversities among the DH lines and hybrids were examined by applying principal coordinate analysis (**Figures 2A, B**). There were 4 subgroups in the DH population, among which POP2 and POP3 were relatively close, possibly because they share a common parent C783 and another parent EH were closely related to UH306. In the hybrid panel, three subgroups were observed, including hybrids using C116A as the tester, Z58 as the tester, DH founders or J2416 as the testers. The LD was estimated for the two panels using SNPs. The LD rapidly decreased with increasing the physical distance between SNPs (**Figure 2C**), but the decay rate varied among the two panels. At r^2 ^= 0.2, the mean LD decay was about 20 Mb and 5 Mb for the DH panel and the hybrid panel.

### 3.3 Genome wide association study

**Figure 3:**
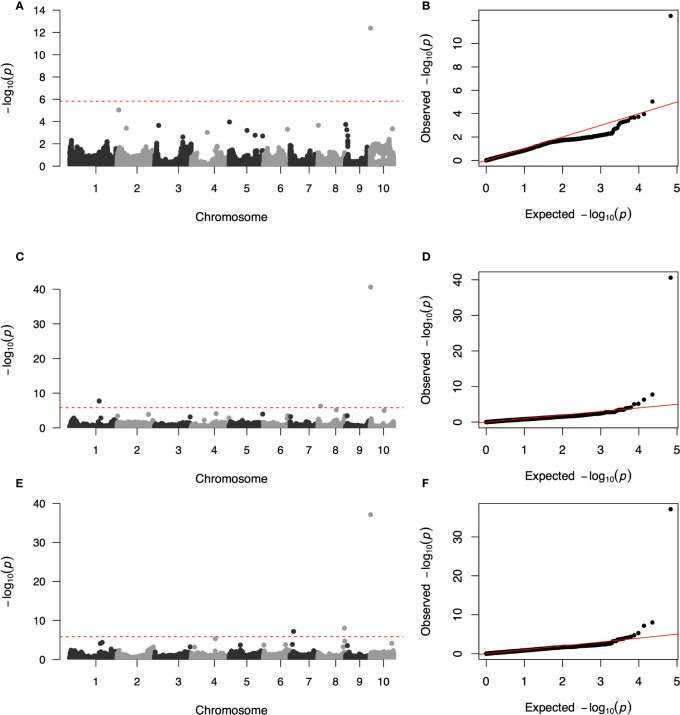
Genome-wide association study Manhattan and quantile–quantile (q–q) plots for Southern Corn Rust (SCR) resistance. **(A, C, E)** Manhattan plots for SCR resistance in additive GWAS in DH panel, additive and dominant GWAS in hybrid panel, respectively. the dashed line corresponds to the threshold level defined at *P* = 1.47 × 10^−6^ by a false discovery rate correction method. **(B, D, F)** q–q plot for SCR resistance in additive GWAS in DH panel, additive and dominant GWAS in hybrid panel, respectively.

Three GWAS processes were performed using the FarmCPU method, including additive GWAS in the DH panel, additive GWAS in the hybrid panel and dominant GWAS in the hybrid panel. The quantile–quantile (q–q) plot implied that the population structure and family relatedness were well controlled in the three GWAS jobs (**Figures 3B, D, F**). One SNP (AX-107958879) on chromosome 10 significantly associated with the SCRRC trait was identified at *P*< 1.47 × 10^−6^ in the DH panel, with effect value was -0.25 (**Figure 3A** and **Table 3**). LD analysis suggested candidate region was 1,150,363–3,990,150 bp, which overlapped the previously reported gene *RPPC* or *RPPK* (**Supplementary Figure 1**). For the additive GWAS in hybrids, three significant SNPs (AX-90698604, AX-108029030, AX-108089672) were detected, with -log10 (*P*) ranging from 6.30 to 40.61. These SNPs were distributed on chromosomes 1, 8 and 10, with the candidate regions Chr1: 181,330,348-188,255,567, Chr8: 13,140,413-18,429,572, Chr10: 2,656,837-4,990,741, respectively (**Figure 3C** and **Table 3**). The effects of them were 0.24, 0.22 and -0.54. For dominant GWAS in the hybrid panel, three SNPs (AX-107981937, AX-108109448, AX-108089672) on chromosomes 7, 8 and 10 significantly associated, with -log10 (*P*) were ranged 7.17-37.12, the effects were -0.16, 0.14 and -0.5 (**Figure 3E** and **Table 3**). Their candidate regions were suggested as Chr7: 13,581,102-23,774,017, Chr8: 167,766,262-168,856,337, Chr10: 2,656,837-4,990,741. The QTL on chromosome 10 obtained by the three GWAS processes were identified as the same region using LD analysis (**Supplementary Figure 1**).

**Table 3:** The results of the genome-wide association (GWAS) analysis.

Type	SNP	Allele	Chromosome	Position	Candidate Intervals		P-value	MAF	Effect
					Left	Rright			
Additive GWAS in DHs	AX-107958879	C/A	10	2,770,918	1,150,363	3,990,150	4.18×10^-13^	0.19	-0.25
Additive GWAS in hybrids	AX-90698604	G/T	1	187,217,509	181,330,348	188,255,567	1.79×10^-8^	0.17	0.24
	AX-108029030	T/C	8	17,058,853	13,140,413	18,429,572	4.83×10^-7^	0.16	0.22
	AX-108089672	C/T	10	3,276,832	2,656,837	4,990,741	2.46×10^-41^	0.18	-0.54
Dominant GWAS in hybrids	AX-107981937	G/A	7	21,288,994	13,581,102	23,774,017	6.74×10^-8^	0.34	-0.16
	AX-108109448	G/A	8	167,766,262	167,766,262	168,856,337	9.46×10^-9^	0.27	0.14
	AX-108089672	C/T	10	3,276,832	2,656,837	4,990,741	7.63×10^-38^	0.18	-0.50

Allele, Letters to the left and right of the “/” refer to major allele and minor allele, respectively.

MAF, minor allele frequency.

### 3.4 Genomic prediction with the different marker density, and training population size

**Figure 4:**
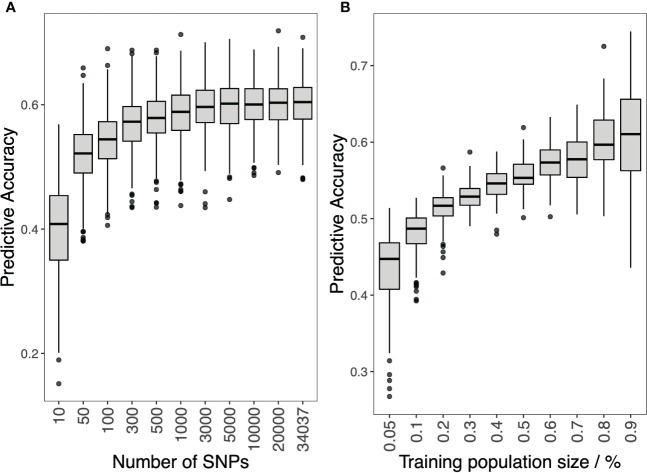
Genomic prediction study in hybrid panel with different SNP numbers **(A)** and training population size **(B)** for Southern Corn Rust (SCR) resistance.

The effect of marker density and training population size on the GP accuracy is shown in **Figure 4**. For marker density, the prediction accuracy increased as the number of markers increased. The prediction accuracy increased rapidly when the number of markers increased from 10 to 5,000. Then, the prediction accuracy increased slightly when the number of markers kept increasing. For training population size, prediction accuracy increased as the size increased, and no slowdown in the growth rate was observed.

### 3.5 Genomic prediction with extended GBLUP models

**Figure 5:**
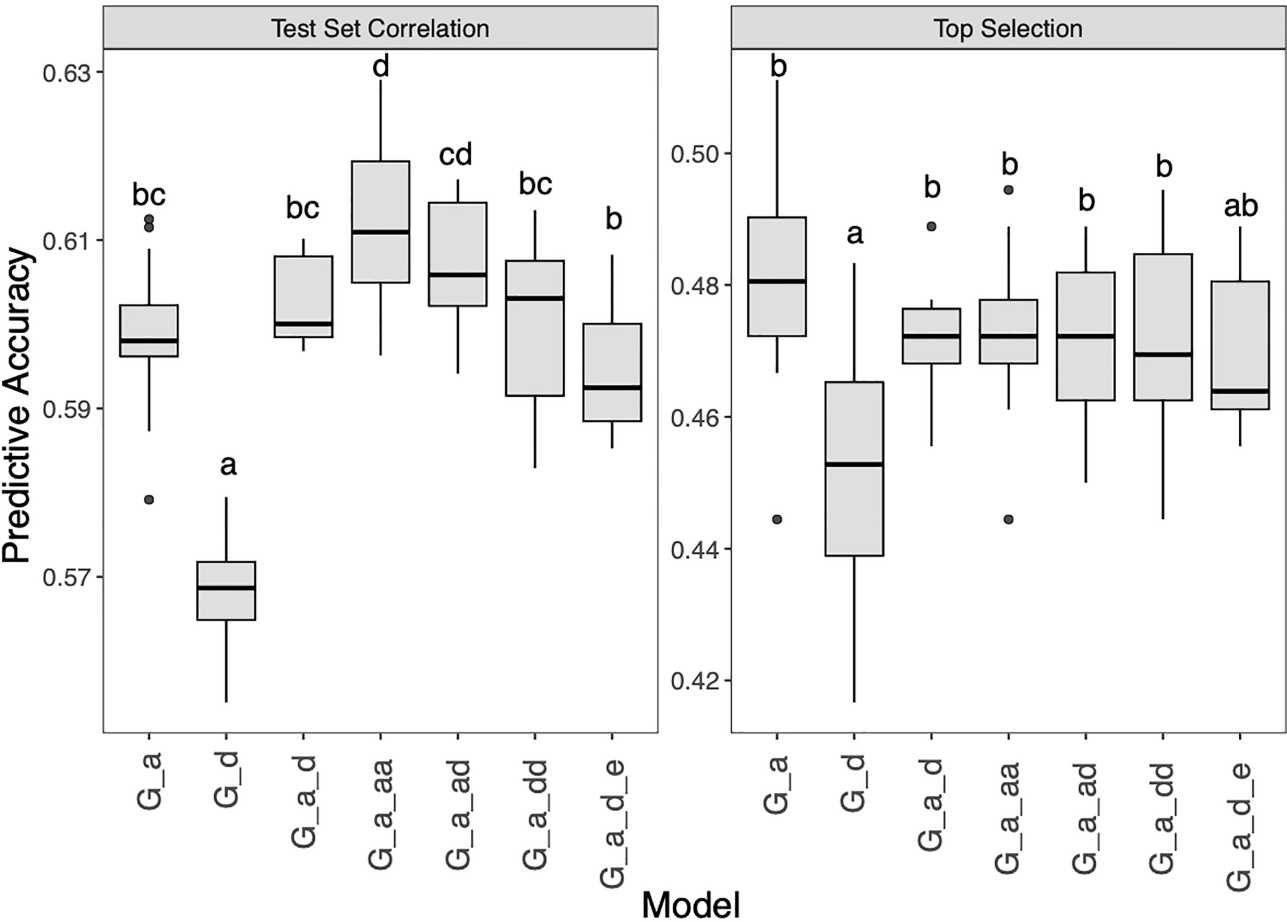
Genomic prediction study in hybrid panel with extend GBLUP models for Southern Corn Rust (SCR) resistance. The left is prediction accuracy for test set and the right is accuracy for top selection.

To meet the breeding needs of SCR-resistant hybrid selection, different GP methods were implemented to improve the prediction accuracy. Firstly, six extended GBLUP models with combinations of additive, dominant, epistatic matrices were tested (**Figure 5** and **Supplementary Table 1**). For test set correlation, the G_A model which only used the additive matrix was found significantly better than the G_D model which used the dominant matrix, with accuracy were 0.60 and 0.57, respectively. Another less accurate model than G_A, but not significant, was the G_A_D_E model, which had a mean accuracy of 0.59. The accuracy of the G_A_AA model was higher than that of the G_A model, suggesting that the epistatic effect was beneficial to GP in this study. Other models (G_A_D, G_A_AD, G_A_DD) performed as well or slightly better than G_A, with the accuracy of 0.60, 0.61 and 0.60, respectively. For top selection accuracy, the overall accuracy was lower than that of the test set correlation. The correlation test shows a significant correlation between the two accuracy evaluation methods, with R=0.77 (*P* value<0.05). Interestingly, G_A is better than other models for top selection, which is different from previous reports (Muñoz et al., 2014). The accuracy of the six models ranged from 0.45 to 0.48, indicating that further improvement is needed.

### 3.6 Genomic prediction with maker effect based models

**Figure 6:**
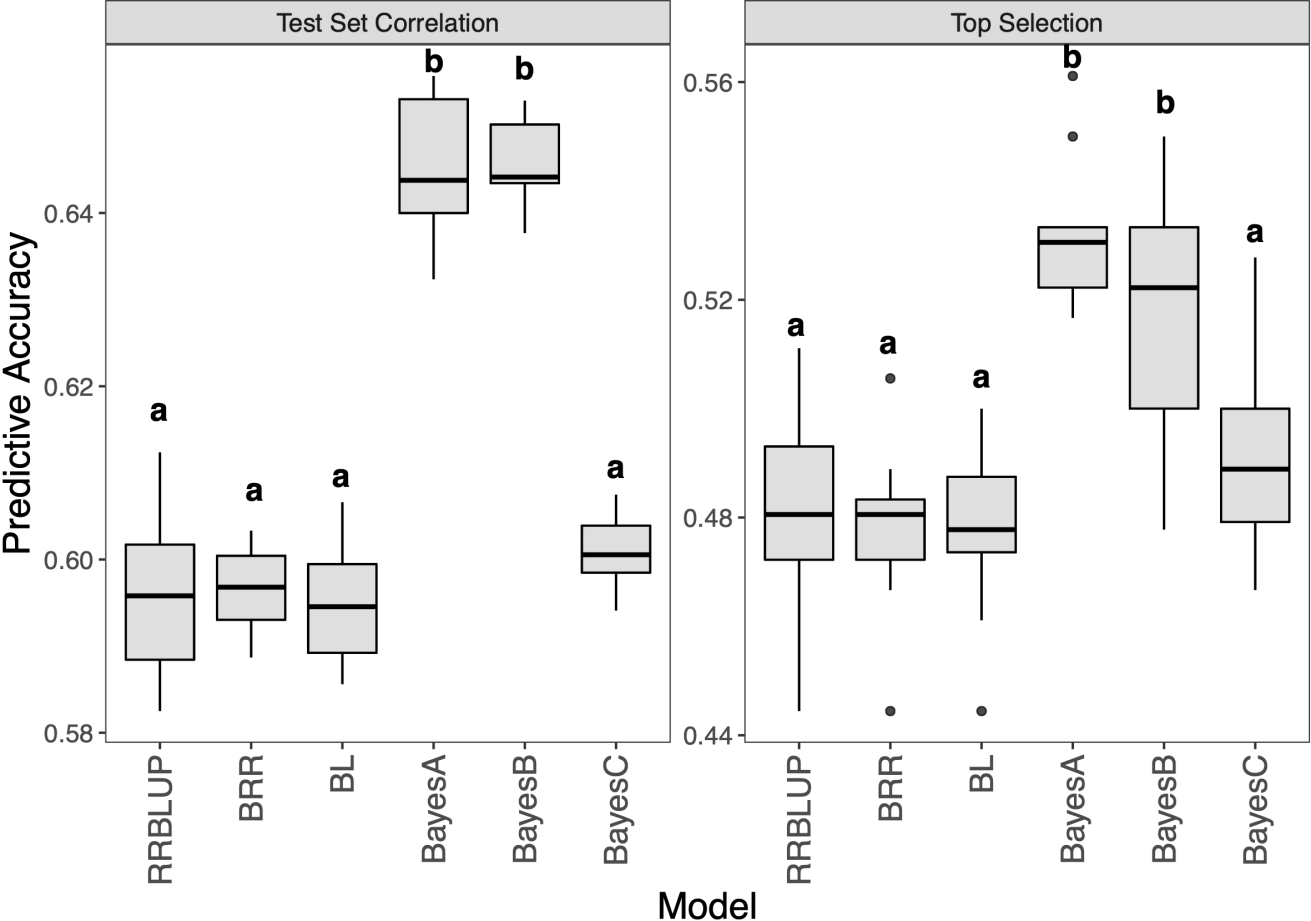
Genomic prediction study in hybrid panel with maker based models for Southern Corn Rust (SCR) resistance. The left is prediction accuracy for test set and the right is accuracy for top selection.

Then, given that our hybrid panel had several significant resistance QTL, six maker based prediction models were performed (**Figure 6** and **Supplementary Table 1**). The results showed that RRBLUP, BRR, BL and BayesC were at the same level with an accuracy of 0.60 for test set correlation. BayesA and BayesB were significantly better than other models with an accuracy of 0.65. The top selection accuracy showed the same trend, the accuracy of BayesA and BayesB were 0.53 and 0.52, respectively, which were significantly higher than other models. Meanwhile, a more significant correlation than extend GBLUP models was detected between the two accuracy evaluation methods (R = 0.98).

### 3.7 Genomic prediction with QTL results

**Figure 7:**
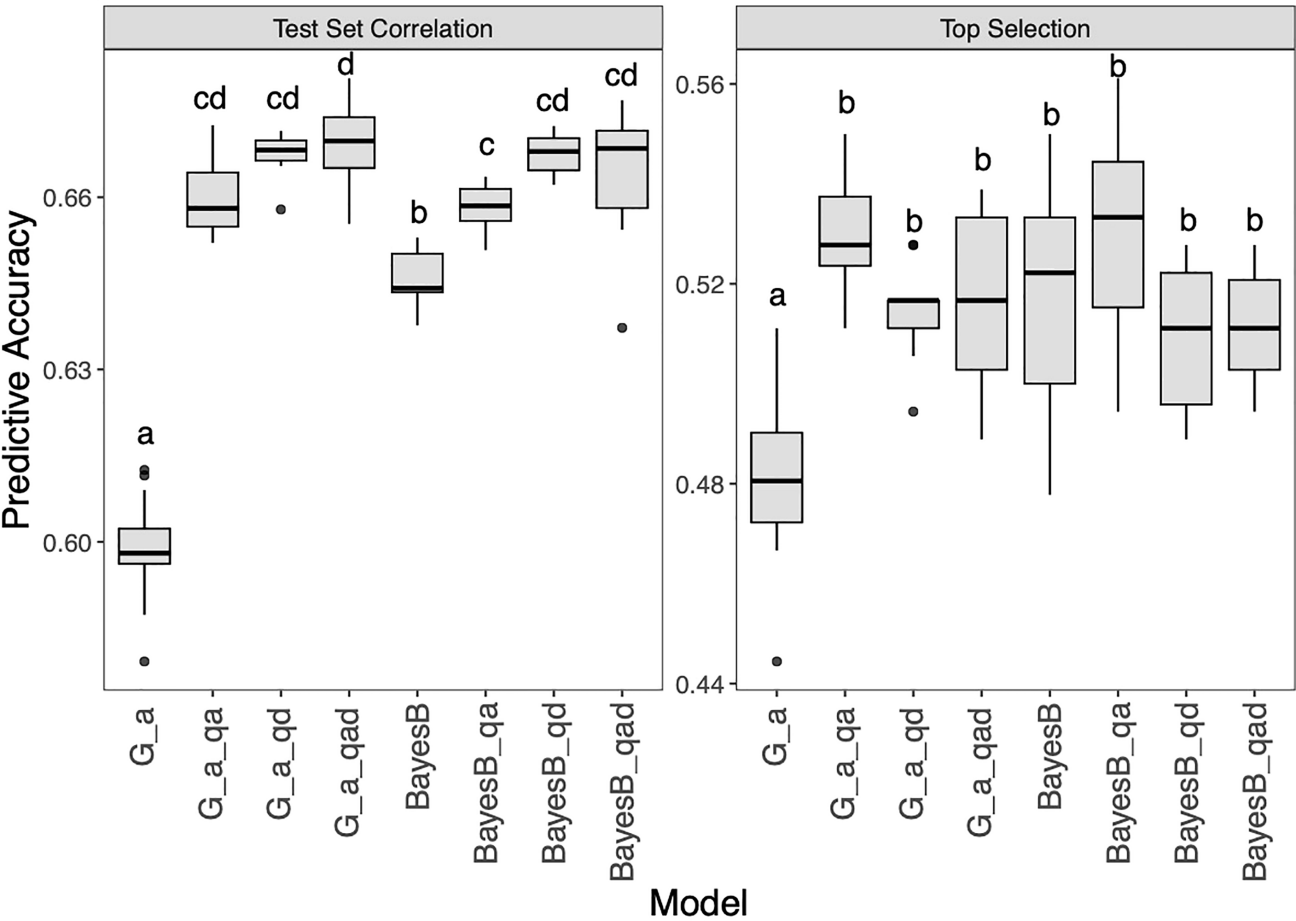
Genomic prediction study in hybrid panel with adding QTL as fixed factor into G_A and BayesB models for Southern Corn Rust (SCR) resistance. The left is prediction accuracy for test set and the right is accuracy for top selection.

Two representative methods (G_A, BayesB) were selected to test the effect of adding QTL as fixation factors (**Figure 7** and **Supplementary Table 1**). For the two models, the GP results showed that the test set correlations were significantly improved whether additive GWAS QTL, dominant GWAS QTL or both were added. The test set correlations for G_A, G_A_qa, G_A_qd, G_A_qad, BayesB, BayesB_qa, BayesB_qd, BayesB_qad were 0.60, 0.66, 0.67, 0.67, 0.65, 0.66, 0.67, 0.66. For top selection using G_A models, the addition of QTL significantly improved the accuracy, among which the G_A_qa model performed best, with an accuracy of 0.55. In contrast, the BayesB model was not significantly changed the accuracy after the QTL addition for top selection accuracy, in which BayesB_qa (0.53) was slightly improved, while BayesB_qd (0.51) and BayesB_qad (0.51) slightly decreased compared with BayesB (0.52).

## 4 Discussion

SCR is a major disease widely existing in maize, which can cause large yield loss and occur in a wider geographical range (Sun et al., 2021). Therefore, it is important to know the genetic basis of rust resistance, and develop appropriate breeding selection strategies. DH technology can shorten time and speed up the breeding process (Ren et al., 2017), so it is popular in modern maize breeding programs. Moreover, due to obtaining the homozygous population quickly, it is also widely used in genetic research (Wang et al., 2012; Shen et al., 2018). Here, we phenotyped SCR resistance in 384 DH lines and 903 testcross hybrids in multi-environment trials. The widely distribution of SCRRS in populations revealed that quantitative genes still played a particularly important role (**Figure 1B**). Comparing the hybrid panel consisting of 12 subgroups, we can find that the SCR resistance of hybrids crossed by C229 was significantly higher than that by Z58 and J2416 (**Table 2**), this is because the genetic contribution of the tester is 50% for each hybrid. This result indicated the importance of the tester in DH-based hybrid breeding, that is, an excellent tester can significantly alter the phenotypic outcome. The heritabilities of DH and hybrid populations were moderate (**Table 2**), suggesting that the SCR resistance was affected by the environment, so the selection of resistant varieties may need to consider regional adaptability.

Unlike the natural line based GWAS analysis, we used the population derived from four bi-parent DH and their testcross hybrid populations. In previous studies, the background of the GWAS homozygous population formed by multiple artificial populations is more controllable, which has been confirmed in the NAM population (Tian et al., 2011; Wu et al., 2016). This method is more powerful than linkage mapping analysis, however, it is also faced with the tight linkage between SNPs, which is not conducive to mapping accuracy. In our study, obvious population structure could be observed in PCA analysis of genotypes (**Figures 2A, B**), but no overfitting was found in GWAS by controlling genetic background (**Figures 3B, D, F**), revealing that these populations can be analyzed by GWAS. At the same time, we found that the LD decay rate could be improved in the hybrid panel (**Figure 2C**), suggesting that for the DH panel, further genetic combination by test-cross could improve the accuracy of GWAS.

It was found that although dominant *Rp* genes mainly functioned in SCR resistance in previous studies, there was evidence that quantitative genes also contributed to SCR resistance (Souza Camacho et al., 2019). Five significant loci were detected in our research (**Figures 3A, C, E**), **Table 3**). DH population only detected one candidate interval, and the number is less than the hybrid panel, which may be due to the larger size or the richer genetic background with the introduced testers in the hybrid populations. In the hybrid panel, three candidate loci were detected by association analysis with additive and dominant coding (**Figures 3C, E** and **Table 3**), two of them were different, which suggested that the dominant effect is also very important in the breeding of rust-resistant hybrids. In all association analyses for 2 populations, a highly significant locus was detected on chromosome 10, which tight chained with the known SCR resistance gene *RppC* (Deng et al., 2022) and other reported QTL, including *RppQ* (Chen et al., 2004), *RppD* (Zhang et al., 2009), *RppS/RppK* (Wu et al., 2015; Chen et al., 2022), *RppM* (Wang et al., 2020). The stability and significant effect of this loci suggested that MAS can be used to fix this region to the germplasm in the breeding process. In addition, four minor genes loci were detected in the hybrid population, and no overlap was found with the known candidate loci, indicating that further fine mapping and function research is needed. Our GWAS analysis enriched the genetic analysis of SCR resistance, demonstrating that many potential SCR resistance genes exist in different maize germplasm backgrounds.

In recent years, GP is a commonly used method to reduce costs and workload in plant and animal breeding programs, especially when combined with DH technology, breeding efficiency can be further improved (Fu et al., 2022). However, for the SCR resistant hybrid selection with GP, experience and reference are lacking. We performed GP analysis on the hybrid panel to explore the prediction accuracy under different GP models. GBLUP, as a classical model of GP, is based on the genetic relationship matrix (Crossa et al., 2017). In hybrid populations, additive, dominant and epistatic effects exist simultaneously. We performed extended GBLUP models and found that the additive-by-additive matrices could significantly improve the prediction performance (**Figure 5**), suggesting that the epistatic effect plays a role in maize SCR resistance. The prediction effect of the pure dominant effect matrix is relatively poor, indicating that the application value of GBLUP only using the dominant matrix is low. It is worth noting that when all matrices were put into the model, the prediction ability is poor, indicating that redundant matrices will reduce the prediction accuracy.

Since the heredity of plant resistance seems to be controlled by dominant genes, GP models based on the genetic relationship matrix may have limited predictive power. We tried maker based GP models and found that most of them had comparable predictive power to G_A, including RRBLUP, BRR, BL and BayesC (**Figure 6**). However, BayesA and BayesB showed a higher prediction ability of 8% beyond other models, which may be due to the difference brought by prior densities of the Bayes model. This difference provided a reference for the prediction of SCR, indicating different GP models significantly impact the prediction power.

Based on the results of GWAS and GP, we further added candidate loci resulting from association analysis into the prediction model as fixed factors (**Figure 7**). The prediction accuracies of the G_A and BayesB models were significantly improved, indicating that QTL information can significantly promote prediction accuracy, which is consistent with previous studies (Jiao et al., 2020). Especially for the G_A model, the prediction accuracy improved by 11.67% after all QTL information was added, which may be due to the G_A_qad model complementing the large effect of QTL on the phenotypic outcome. In breeding applications, prediction ability can be improved by adding known QTL loci to GP models. In addition, implementing GP in hybrids is more complex than in homozygous populations, and it may be more efficient to explore a combination of multiple approaches.

## 5 Conclusion

SCR occurs widely in maize and brings great loss to yield. Here, we developed the DH panel with 384 lines and the hybrid panel with 903 testcross hybrids. SCRRS of accessions were collected with multi-year and multi-location field testing. Using GWAS analytical pipeline, five QTL loci were detected on chromosomes 1, 7, 8, 8, and 10, with *P*-values ranging from 4.83×10^-7^ to 2.46×10^-41^. On the other hand, to improve the selection efficiency of resistant materials in breeding, several GP methods were performed to explore predictive ability for SCRRS in hybrids, including extended GBLUP with different genetic matrices, maker based prediction models, and mixed models with QTL as fixed factors. We found that adding additive-by-additive effect to GBLUP model, selecting BayesA or BayesB model, adding QTL into the mixed linear prediction model will improve the prediction performance. The results will provide important valuable information for understanding the genetic architecture and the application of GP for SCR in maize breeding.

## Data availability statement

The original contributions presented in the study are included in the article/**Supplementary Material**. Further inquiries can be directed to the corresponding authors.

## Author contributions

JL and DC: investigation, data processing, and writing. SG, CC, YuwW, YZ, XQ, ZL, and DW: investigation, data collection and editing. WL and YuaW: managing the project, review and editing. CL and SC: managing the project, editing the manuscript, and funding acquisition. All authors contributed to the article and approved the submitted version.

## Supplementary material

Supplementary Figure 1 Linkage disequilibrium (LD) heatmap around the SNPs derived from genome-wide association (GWAS) analysis on chromosome 10.
