# Supplementary figures and images for: Genome-wide association and genomic prediction for resistance to southern corn rust in DH and testcross populations
Source: Front Plant Sci. 2023 Jan 26;14:1109116. doi: 10.3389/fpls.2023.1109116 (PMC9908600; doi:10.3389/fpls.2023.1109116)

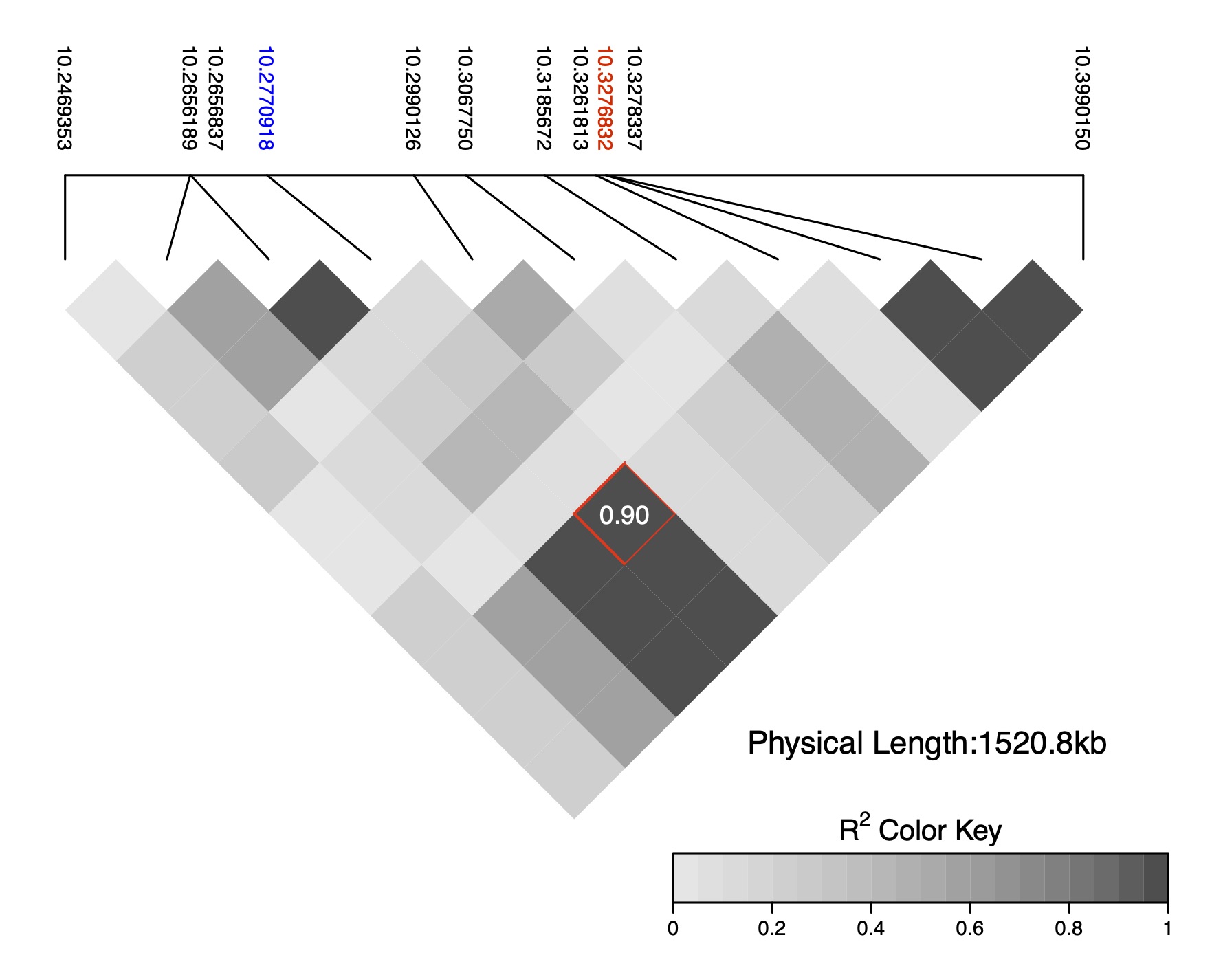

Supplement: Supplementary Figure 1 — Linkage disequilibrium (LD) heatmap around the SNPs derived from genome-wide association (GWAS) analysis on chromosome 10. [file Image_1.jpeg]
